# Nontuberculous mycobacterial disease in children: A systematic review and meta-analysis

**DOI:** 10.1016/j.heliyon.2024.e31757

**Published:** 2024-05-22

**Authors:** Xiaoyi Jiang, Yi Xue, Peixuan Men, Liping Zhao, Junnan Jia, Xia Yu, Hairong Huang

**Affiliations:** a National Clinical Laboratory on Tuberculosis, Beijing Key Laboratory for Drug-resistant Tuberculosis Research, Beijing Chest Hospital, Capital Medical University, Beijing Tuberculosis and Thoracic Tumor Institute, Beijing, China; b Institute of Medical Information/Medical Library, Chinese Academy of Medical Sciences and Peking Union Medical College, Beijing, 100005, China

**Keywords:** Child, Nontuberculous mycobacteria, Clinical lesion sites, Species

## Abstract

**Background:**

The prevalence of nontuberculous mycobacterial (NTM) disease in children is increasing worldwide. The clinical manifestations of pediatric NTM patients are significantly different from those of adult patients, but the knowledge of the disease is generally poor.

**Methods:**

English databases (PubMed, Web of Science, Embase, BIOSIS) and Chinese databases (CNKI, Wanfan, VIP) were searched on October 15th, 2022. All the articles of cross-sectional and cohort studies reporting the species composition and lesion site of the NTM disease in children using well-recognized NTM species identification methods were taken into account. Using a random effects model, we assessed the disease lesion sites and the prevalence of different NTM species in pediatric NTM disease. Sources of heterogeneity were analyzed using Cochran's Q and the I^2^ statistic. All analyses were performed using CMA V3.0.

**Results:**

The prevalence rates of NTM disease in children ranged between 0.6 and 5.36/100,000 in different countries, and Europe reported the highest prevalence rate. The most common clinical lesion site was lymph node, accounting for 71.1 % (55.0 %–83.2 %), followed by lung (19.3 %, 9.8%–34.4 %）and then skin and soft tissue (16.6 %,13.5%–20.3 %). *Mycobacterium avium* complex (MAC) was the most isolated NTM pathogen in children, accounting for 54.9 % (39.4%–69.6 %). Inconsistent with adult patients, *Mycobacterium avium* accounted for a dominant proportion in MAC than *Mycobacterium intracellulare*.

**Conclusions:**

The lymph node was the most affected organ in pediatric NTM disease, while *Mycobacterium avium* was the most isolated pathogenic species in children.

## Highlights

• The comprehensive feature of nontuberculous mycobacterial diseases in children is addressed for the first time in this article.
• The most common clinical lesion site was lymph node, accounting for 71.1 % (55.0%–83.2 %).
• *Mycobacteriumavium* complex (MAC) was the most isolated NTM pathogen in children.

## 1 Introduction

Nontuberculous mycobacteria (NTM) is a generic term used for mycobacterial species except *Mycobacterium tuberculosis* complex (MTBC) and *Mycobacterium leprae*. NTM is ubiquitous in the surroundings and is recognized as an opportunistic pathogen, which means that it is more likely to colonize in the host's respiratory tract and seldom cause disease. However, NTM can be more virulent for the immunocompromised individuals. In recent years, an increased incidence of NTM-related diseases has been reported in adults [1,2]. In many developed countries, such as the United States, France, and the Netherlands, the NTM disease case has surpassed tuberculosis (TB) [3]. A concomitant increase of NTM disease in children was also observed [4], which has been suggested to be related to abrogation of BCG vaccination and some specific living styles [5].

NTM is categorized into rapidly growing mycobacteria (RGM) and slowly growing mycobacteria (SGM), according to the time needed to get visible colony growing on solid medium. The primary disease caused by NTM in adults is lung infection, which incurs symptoms resembling tuberculosis, such as cough, weight loss, and fatigue. Rarely, NTM pulmonary disease also results in respiratory failure. Some RGM species could cause skin abscesses, cellulitis, and other skin infections, usually due to a trauma or surgical procedure. In immune-compromised individuals, such as those with HIV/AIDS, NTM can cause disseminated infections, potentially affecting various organs, including the liver, spleen, and bone marrow.

NTM disease in children is relatively underreported and the clinical manifestations differ from those in adults [6,7], NTM disease in children most frequently affects lymph nodes, especially the cervical lymph node [4]. Disease in the lungs and skin could also happen in children but with a much lower chance. To diagnose NTM disease of lymph node, an invasive way to collect lymph tissue for bacterial examination is needed, which is hard to implement. Furthermore, the existing techniques for identifying mycobacterial species are limited and not easily accessible. Therefore, delayed diagnosis in pediatric NTM disease is very common. Moreover, the less awareness of the disease among health professionals is another key reason for the delayed diagnosis of NTM disease in children.

In this article, based on a meta-analysis of the literature on pediatric NTM disease published before October 15, 2022, we comprehensively described the incidence rate, demographic characteristics, common clinical manifestations, and the causative species of pediatric NTM diseases. We also conducted a brief comparison of patient characteristics across different continents. Since no such meta-analysis on pediatric NTM disease has been conducted previously, we believe that the data we presented herein would benefit a better understanding of this very-ignored childhood disease.

## 2 Methods

### 2.1 Search strategies

The English databases searched included PubMed, Web of Science, Embase, and BIOSIS. The Chinese databases searched included CNKI, Wanfan, and VIP. The closing date of the search was October 15, 2022. The terms included "non-tuberculosis", "non-tuberculous", "non-tuberculous mycobacterium", "non-tuberculous mycobacteria", "NTM", "MOTT", "atypical mycobacterium", "infant", "infants", "newborn", "newborns", "neonate", "neonates", "toddler", "toddlers", "pediatric", "pediatrics", "preschooler", "children", "child" and other keyword combinations to screen related research. Additional studies were also searched using a cross-referencing strategy.

### 2.2 Inclusion and exclusion criteria

We screened and selected articles according to the preferred reporting items of the systematic review and Meta-Analysis (PRISMA) 2020 Statement guidelines [8], taking into account all the articles of cross-sectional and cohort studies reporting the species composition and lesion site of the NTM disease in children using a well-recognized NTM species identification methods. The acceptable species identification methods included mass spectrometry or molecular methods, such as DNA sequencing, PCR restriction fragment length polymorphism (RFLP) assay, Multi-locus sequence typing, Reverse dot blot technique, and metagenomic next-generation sequencing.

The study was excluded from the analysis due to any of the following reasons: focused on only one NTM disease type (lymph node disease, lung disease or Buruli ulcer); focus on only one type of bacteria, such as *M. avium* or *M. abscessus*; only involved a specific patient population, such as patient with previous history of TB treatment; review article, conference report, case report or study reported in languages other than Chinese or English; study with less than 10 cases. In cases where the study included patients aged 18 years or older, we selectively enrolled only those patients under the age of 18, provided that they could be distinctly distinguished from the older patient group and that all relevant information of interest was accessible.

### 2.3 Selection of studies for review

All articles identified from bibliographic databases were exported to *NoteExpress* (V3.0, Aegean Sea, Beijing, China) for reference management. After removing duplicates, two authors (J.X and X.Y) independently screened articles based on title and abstract. Subsequently, the two authors (J.X and X.Y) independently evaluated the full texts of the included articles by strictly applying the inclusion and exclusion criteria. Any disagreements between the two authors during the process were resolved through discussion and consensus.

### 2.4 Data extraction and definitions

For all the studies, we extracted the following metadata from the included publications: first author, year of publication, study location, study duration, number of investigated cases, patient age, study methodology, sample source and size, prevalence of NTM infection in children, species composition, and disease type. Two investigators (J.X and M.P.) searched the databases to identify the literature and extracted the data from them independently. A third investigator (X.Y.) checked the findings of these two investigators. The inconsistencies among the reviewers in either the decision or inclusion of studies or data extraction were discussed to obtain consensus.

### 2.5 Quality assessment

For cross-sectional studies, the evaluation used the standards recommended by the Agency for Healthcare Research and Quality (AHRQ), which consist of 11 items with a maximum score of 11. A score of 0–3 is considered a low-quality study, 4–7 is a moderate-quality study, and ≥8 is a high-quality study. The quality of the studies was independently assessed by two researchers (Z.L and J.J). Any differences that arose during the process were discussed to reach a consensus.

### 2.6 Statistical analysis

A meta-analysis was performed using comprehensive meta-analysis (V3.0, Biostat, Englewood, NJ, USA) regarding the pathogenic bacterial species and the lesion sites of the NTM disease in children and their 95 % confidence intervals (95 % CI). Random effect models were used and tested with Cochran's Q and I^2^ statistics taking into account the possibility of heterogeneity between studies. Publication bias was statistically assessed using the Egger weighted regression method (P < 0.05 was considered to indicate statistically significant publication bias).

## 3 Results

### 3.1 Characteristics of the included studies

**Fig. 1:**
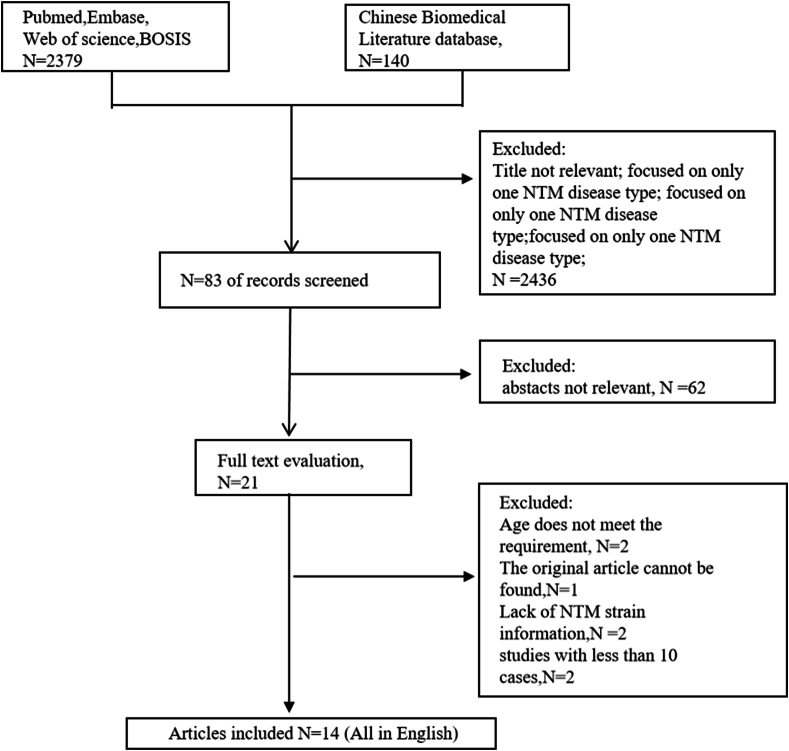
Flow diagram of study identification.

With the given terms in different combinations, a total of 2379 English literatures and 140 Chinese literatures were searched (Fig. 1). After double screenings and duplication inspection, 21 articles underwent full-length evaluation. Seven articles were then excluded due to non-compliance. Finally, a total of 14 articles fully qualified the inclusion criteria [5,[9], [10], [11], [12], [13], [14], [15], [16], [17], [18], [19], [20], [21]]. These articles, all published in English, collectively involved 1352 NTM isolates. We summarized the main data of these studies in Table 1. According to the AHRQ's quality evaluation standard, four of the 14 cross-sectional studies met the criteria for high-quality, while the rest were rated as moderate-quality.(Table 2).

**Table 1:** Characteristics of studies involved in the current systematic review and meta-analysis.

First author	Time of study	Publication date	Nation	Age range	Median age (years)	Incidence rate	NTM isolations
Blyth et al. [9]	2004.07–2007.06	2009	Australia	1.1–14.2 years old	2.9	0.84/100000	68
Reuss et al. [10]	2003.04–2005.09	2009	Germany	≤15 years old	2.5	3.1/100000	101
Cruz et al. [11]	2003.06–2008.06	2010	America	2 months-17 years old	2.7	/	75
Pham-huy et al. [12]	2005.09–2006.08	2010	Canada	<18 years old	/	2.15/100000	29
Asiimwe et al. [5]	2009–2011	2013	Uganda	4–11months old and12-16 years old	/	/	63
Haverkamp et al. [13]	2001.04–2003.04	2015	Netherlands	6 months-12.6 years old	2.7	0.77/100000	27
Irohtam et al. [14]	2010–2013	2015	America	10 months-17years old	11	/	39
Tebruegge et al. [15]	2000–2016	2016	Australia	0–14 years old	2.8	0.6–1.6/100000	90
Laila et al. [16]	2005.07–2015.06	2016	Australia	1.9–14.7 years old	2.2	0.84/100000	54
Termansen et al. [17]	1991–2015	2017	Denmark	0–14 years old	2.5	5.36/100000	561
Kontturi et al. [18]	1995–2016	2018	Finland	4–59 months old	2.3	3.9/100000	97
Varghese et al. [19]	2016–2017	2019	Saudi Arabia	0–14 years old	/	/	52
Kaguthi et al.[20]	2009.06–2010.06	2019	Kenya	<23 months old	/	/	16
Dolezalova et al. [21]	2011–2018	2020	Czech Republic	/	2	0.53/100000	80

**Table 2:** Quality assessment of the included cross-sectional studies.

Item	Blyth et al.	Reuss et al.	Cruz et al.	Pham-huy et al.	Asiimwe et al.	Haverkamp et al.	Irohtam et al.	Tebruegge et al.	Laila et al.	Termansen et al.	Kontturi et al.	Varghese et al.	Kaguthi et al.	Dolezalova et al.
1) Define the source of information (survey, record review)	1	1	1	1	1	1	1	1	1	1	1	1	1	1
2) List inclusion and exclusion criteria for exposed and unexposed subjects (cases and controls) or refer to previous publications	0	1	0	1	0	1	1	1	1	1	0	0	0	0
3) Indicate time period used for identifying patients	1	1	1	1	1	1	1	1	1	1	1	1	1	1
4) Indicate whether or not subjects were consecutive if not population-based	1	1	1	1	1	1	1	1	1	1	1	1	1	1
5) Indicate if evaluators of subjective components of study were masked to other aspects of the status of the participants	0	1	0	1	0	1	0	0	0	0	0	0	0	0
6) Describe any assessments undertaken for quality assurance purposes (e.g., test/retest of primary outcome measurements)	1	1	1	0	1	1	0	1	1	1	1	1	1	1
7) Explain any patient exclusions from analysis	1	1	1	1	0	1	1	1	1	0	1	1	0	0
8) Describe how confounding was assessed and/or controlled	0	1	0	1	0	1	0	1	0	0	1	0	0	0
9) If applicable, explain how missing data were handled in the analysis	1	0	0	1	0	0	0	0	0	0	0	0	0	0
10) Summarize patient response rates and completeness of data collection	1	1	1	1	1	1	1	1	1	0	1	1	1	0
11) Clarify what follow-up, if any, was expected and the percentage of patients for which incomplete data or follow-up was obtained	0	0	0	0	0	0	0	0	0	0	0	0	0	0
**Total**	7	9	6	9	5	9	6	8	7	5	7	6	5	4

### 3.2 Epidemiological feature of NTM disease in children

Among the 14 studies, 9 reported the prevalence rates of NTM disease in children, which ranged between 0.6 and 5.36/100,000. The highest rate was reported from Denmark, followed by another two European countries Finland and Germany. In contrast, the Netherlands, also located in Europe, reported a relatively lower prevalence rate of 0.77/100,000. The reported susceptible age of NTM disease in children from different countries consistently ranged between 2 and 3 years old, albeit a single article [14] from a hospital in the United States reported a median age of 11. Patient gender was recorded in 9 out of the 14 articles, including 716 individuals. Females accounted for 52.0 % (371/716) and males were 48.0 % (345/716). There was no significant difference considering the gender of the NTM patients (P＞0.05)（Table 3）.

**Table 3:** Gender analysis and disease lesion sites of NTM disease in children.

	Subgroup	No.of study	Proportion (95 % CI)	n/N	Heterogeneity I^2^	Heterogeneity,P value	Egger's test, t	Egger's test, P value
**Gender**	Male	9	48.0 (42.2–53.9)	345/716	57.035	0.017	0.329	0.376
	Female	9	52.0 (46.1–57.8)	371/716	57.035	0.017	0.329	0.376
**Lesion sites**	Lymph node disease	8	71.1(55.0–83.2)	440/631	92.078	＜0.001	1.348	0.226
	Pulmonary disease	7	19.3（9.8–34.4）	92/793	91.051	＜0.001	2.142	0.085
	Skin and soft tissue infection alone	6	16.6（13.5–20.3）	76/468	0	0.445	1.803	0.146
	Bacteremia	4	10.0（5.4–17.9）	27/283	60.802	0.054	3.554	0.071
	Genitourinary	1	1.9（0.3–12.4）	1/52	/	/	/	/
	Peritoneum	1	3.8（0.1–14.1）	2/52	/	/	/	/

### 3.3 Disease lesion sites in pediatric NTM disease

Eight out of the 14 articles analyzed the lesion sites of NTM disease in children. Meta-analysis demonstrated that 71.1 % (55.0%–83.2 %) of the patients presenting lymph node disease, 19.3 % (9.8%–34.4 %) lung disease, 16.6 % (13.5%–20.3 %) had skin and soft tissue disease, and 10 % indicated bacteremia. Only one study reported cases of peritoneum disease and genitourinary disease, which accounted for 3.8 % (0.1%–14.1 %) and 1.9 % (0.3%–12.4 %) of the total 52 patients, respectively (Table 3).

### 3.4 Prevalence of different NTM species in pediatric NTM disease

A total of 1352 isolates, including 26 different species, were reported in the 14 included articles. These isolates encompass 14 slow-growing mycobacteria (SGM) species and 12 rapid-growing mycobacteria (RGM) species (Table 4). Among the SGM species, *M. avium* showed the highest prevalence by accounting for 45.1 % of the isolates (with a range of 18.5 %–74.8 %). Following was *M. szulgai,* which constituted 14.3 % of the isolates (with a range of 7.6 %–25.3 %). *M. fortuitum* and *M. immunogenum* were the most frequently isolated RGM in pediatric NTM disease patients with rates of 10.3 % for both. However, *M. immunogenum* was reported in only one article from the USA [14]. Further analysis focused on *Mycobacterium avium* complex (MAC), which mainly consists of *M. avium* and *M. intracellulare,* and *M. chelonae-M. abscessus* group as well. MAC accounted for a dominant proportion in SGM, with a 54.9 % (39.4%–69.6 %) isolation rate. *M. avium* had a significantly higher percentage (45.1 %,18.5%–74.8 %) than that of *M. intracellulare* (4.7 %, 3.0%–7.5 %)(P = 0.02). The *M. chelonae-M.abscessus* group accounted for 13.7 % (7.0%–24.8 %) of the RGM isolates. Among the 7 articles that categorized *M. chelonae-M. abscessus* group into *M. chelonae* and *M. abscessus* species separately, M. abscessus had a slightly higher but not statistically significant percentage (6.1 %,2.4%–14.5 %) than that of *M. chelonae* (4.8 %,1.1%–19.0 %))(P = 0.11).

**Table 4:** Analysis of pathogenic bacteria of pediatric NTM disease.

Classification	species	No. of study	Prevalence of NTM (95 % CI)	n/N	Heterogeneity I^2^	Heterogeneity,P value	Egger's test, t	Egger's test,P value
**Slowly growing mycobacteria**	*MAC*	14	54.9（39.4–69.6）	910/1352	94.167	<0.001	2.093	0.029
	*M. avium	7	45.1（18.5–74.8）	252/412	91.35	<0.001	1.495	0.098
	*M. szulgai*	1	14.3（7.6–25.3）	9/63	/	/	/	/
	*M. ulcerans*	2	11.6（7.3–17.9）	17/158	63.997	0.096	/	/
	*M. malmoense*	3	6.3（3.3–11.7）	9/146	0	0.955	0.07	0.096
	*M. gordonae*	4	5.5（2.5–11.9）	11/231	34.637	0.204	2.051	0.089
	*M. lentiflavum*	4	5.3（3.1–9.0）	13/294	0	0.669	2.882	0.102
	*M. intracellulare	7	4.7（3.0–7.5）	16/412	0	0.426	1.973	0.053
	*M. simiae*	5	4.4（0.9–18.7）	24/373	88.4	<0.001	3.042	0.056
	*M. paratuberculosis*	1	3.7（0.5–2.2）	’1/27	/	/	/	/
	*M. marinum*	3	3.2（1.5–7.0）	6/187	0	0.987	0.117	0.926
	*M. kansasii*	3	3.0（1.2–7.5）	7/250	21	0.282	3.379	0.183
	*M. haemophilum*	1	2.9（0.7–1.1）	2/68	/	/	/	/
	*M. scrofulaceum*	3	2.5（0.9–6.4）	4/187	0	0.583	0.500	0.352
	*M. terrae*	1	1.1（0.2–7.5）	1/90	16	/	/	/
**Rapid growing mycobacteria**	*M. chelonae-M. abscessus group*	9	13.7（7.0–24.8）	70/457	83.854	<0.001	1.975	0.044
	*M. fortuitum*	10	10.3（4.3–22.8）	85/603	90.222	<0.001	2.591	0.016
	*M.immunogenum*	1	10.3（3.9–25.3）	4/39	/	/	/	/
	*M. celatum*	2	6.3（2.0–17.8）	3/48	0	0.82	/	/
	*M. abscessus	7	6.1（2.4–14.5）	22/314	71.157	0.004	4.266	0.006
	*M.monacense*	1	5.8（1.9–16.4）	3/52	/	/	/	/
	*M. chelonae	7	4.8（1.1–19.0）	22/314	68.020	0.005	4.676	0.003
	*M. mucogenicum*	1	3.8（1.0–14.1）	2/52	/	/	/	/
	*M.riyadhense*	1	3.8（1.0–14.1）	2/52	/	/	*/*	/
	*M. peregrinum*	2	2.9（0.7–10.8）	2/114	0	0.39	/	/
	*M. interjectum*	2	2.4（0.8–7.3）	3/116	0	0.671	/	/
	*M. kubicae*	1	1.9（0.3–12.4）	1/52	/	/	/	/
	*M. gilvum*	1	1.1（0.2–7.5）	1/90	/	/	/	/

## 4 Discussion

Pediatric NTM disease is a globally neglected health concern, as it demands the expertise of knowledgeable physicians and access to advanced laboratory techniques for detecting NTM bacteria and identifying specific species. Unfortunately, meeting both of these requirements is challenging for the majority of countries around the world. We identified only 14 qualified articles from 11 countries that included data on lesion sites and/or pathogenic NTM species. Generally, each country only had one or two publications, except Australia which had three. Considering the continental origin, even though Europe had the highest number of publications, the total was only 5. Saudi Arabia presented the only eligible publication from Asia [19], while none was from South America. This condition underscores the overall lag in the diagnosis of pediatric NTM disease worldwide.

Out of the identified publications, only nine included prevalence data of pediatric NTM disease which varied significantly, ranging between 0.6/100,000 and 5.36/100,000. Notably, a study conducted in Finland [18] revealed a markedly lower incidence of pediatric NTM disease in the universal BCG vaccination group (0.2 per 100,000 person-years) than in the selective BCG vaccination group (3.9 per 100,000 person-years). This observation implies that the prevalence of NTM disease in children may be influenced by BCG vaccination. Two studies presented the correlational data of age and lesion site of the enrolled patients and found that the median age of patients with lymph node disease was generally lower than that of children with lung or skin and soft tissue disease [12,15].

In terms of lesion sites of the disease, unlike adults who mainly develop lung diseases [22], 71.1 % of the included pediatric NTM patients had lymph node diseases, followed by 19.3 % lung diseases and 16.6 % skin and soft tissue disease cases. This evident difference between adults and children in the infected organs may reflect the differences in the route of infection and the strength of the immune system. It was suggested that children are infected with NTM, after contact with bacterial soil and water, through the mouth and then the bacteria colonize in lymph nodes [23], whereas adults are more likely to be infected by inhalation through the respiratory tract. In addition, children's immune system is less competent, and their lymph nodes are immature [24,25]. The exact mechanism that causes this big difference in infected organs between children and adults remains unknown, but some factors leave children prone to the disease. A few articles identified that a significant proportion of children with NTM pulmonary diseases had underlying lung diseases, with cystic fibrosis being the most common precondition [12,14,16]. Moreover, individuals with cystic fibrosis tended to contract NTM infections at advanced age compared to those without [11]. Certain studies documented that the majority of children with NTM bacteremia had episodes related to central venous catheter use^.^ [9,11,16] Furthermore, children with soft tissue or bone marrow infections frequently had a recent history of puncture injuries [16].

In our analysis of the most common species causing NTM diseases in children, we found that among SGM, MAC was the most isolated pathogenic NTM, accounting for 54.9 % of the cases, followed by *M. szulgai* at 14.3 %. The most frequently isolated RGM species was *M. chelonae-M. abscessus* group (13.7 %). These species constitution characteristics are similar to adult patients who mainly have lung diseases. However, the species constitution within MAC and *M. chelonae-M. abscessus* group seemed very different from those of the adult patients. While *M. intracellular*e accounted for the absolute majority of MAC in adult patients [1], *M. avium* is dominated in MAC in pediatric patients. Furthermore, *M. abscessus* accounts for a much bigger proportion of *M. chelonae-M. abscessus* group in adult patients, whereas no significant difference was observed considering the proportions of these two species in pediatric patients in this study.

The biggest limitation of this meta-analysis is the limited data retrieved from publications. This flaw reflects that there are many barriers to diagnosing pediatric NTM disease in clinical practice, and the current prevalence might be underestimated. Furthermore, the substantial heterogeneity of NTM diseases among countries, including variations in prevalence rates and species constitutions, may introduce significant bias when analyzing the data from only a few countries. Thirdly, although the majority of the 14 included articles reported outcomes of nationwide studies, a few were confined to a specific region or a given hospital. Therefore, this less representativeness would further introduce some more bias. However, the goal of this study was to increase the awareness of healthcare providers on this disease, even though the pooled percentages might not so accurately reflect the real condition.

In summary, this systematic review and meta-analysis identified that pediatric NTM disease manifests very different features from adult patients. In contrast to lung disease in adult patients, the lymph node was the most affected organ in children. Although MAC is the most isolated pathogen for NTM disease both in children and in adults, *Mycobacterium avium,* rather than *Mycobacterium intracellulare,* dominated the species constitution of MAC in children [26]. A better understanding of the NTM disease would help to combat this troublesome disease which is increasing worldwide in both children and adults.

## Conflict of interest disclosures

The authors declare no conflict of interest in this work.

## Funding source

This study was supported by research funding from the 10.13039/100014718Natural Science Fund of China (82072328), Beijing Public Health Experts Project (grant number G2023-2-002 and G2023-3-004); Beijing Hospitals Authority Youth Program (QML20211602) and Beijing Nova Program (20230484411). The Foundation had no role in the conduct of the review.

## Availability of data and materials

All data generated or analyzed during this study are included in this published article.

## Ethical approval statement

This systematic review does not require ethical approval according to the international standards for Good Clinical Practice.

## Consent for publication

Not applicable.

## CRediT authorship contribution statement

**Xiaoyi Jiang:** Writing – review & editing, Writing – original draft, Software, Project administration, Data curation, Conceptualization. **Yi Xue:** Supervision, Methodology, Investigation, Data curation. **Peixuan Men:** Investigation, Data curation, Conceptualization. **Liping Zhao:** Visualization, Validation, Methodology, Data curation. **Junnan Jia:** Methodology, Investigation, Formal analysis. **Xia Yu:** Writing – review & editing, Visualization, Supervision, Methodology, Formal analysis. **Hairong Huang:** Writing – review & editing, Supervision, Methodology, Investigation, Funding acquisition, Formal analysis.

## Declaration of competing interest

The authors declare that they have no known competing financial interests or personal relationships that could have appeared to influence the work reported in this paper.
